# Lack of association between toll-like receptor 4 gene polymorphisms and sarcoidosis-related uveitis in Japan

**Published:** 2009-12-10

**Authors:** Yuri Asukata, Masao Ota, Akira Meguro, Yoshihiko Katsuyama, Mami Ishihara, Kenichi Namba, Nobuyoshi Kitaichi, Shin-ichiro Morimoto, Toshikatsu Kaburaki, Yasutaka Ando, Shinobu Takenaka, Hidetoshi Inoko, Shigeaki Ohno, Nobuhisa Mizuki

**Affiliations:** 1 Department of Ophthalmology and Visual Science, Yokohama City University Graduate School of Medicine, Yokohama, Kanagawa, Japan; 2 Department of Legal Medicine, Shinshu University School of Medicine, Matsumoto, Nagano, Japan; 3 Department of Pharmacy, Shinshu University Hospital, Matsumoto, Nagano Japan; 4 Hiyoshi Eye Clinic,Yokohama, Japan; 5 Department of Ophthalmology and Visual Science, Hokkaido University Graduate School of Medicine, Sapporo, Hokkaido, Japan; 6 Division of Cardiology, Department of Internal Medicine, Fujita Health University School of Medicine, Toyoake, Aichi, Japan; 7 Department of Ophthalmology, University of Tokyo School of Medicine, Tokyo, Japan; 8 Department of Ophthalmology, Kitasato Institute Hospital, Tokyo, Japan; 9 Department of Respiratory Diseases, Kumamoto City Hospital, Kumamoto, Japan; 10 Department of Basic Science and Molecular Medicine, Tokai University School of Medicine, Isehara, Kanagawa, Japan

## Abstract

**Purpose:**

Toll-like receptors (TLRs) are pattern-recognition receptors that play an important role in innate and adaptive immune responses to microbial pathogens. Among TLRs, TLR4 recognizes lipopolysaccharides of Gram-negative bacteria. Genetic polymorphisms within the *TLR4* gene have been reported to be associated with various inflammatory diseases; therefore, *TLR4* appears to be a susceptibility gene for sarcoidosis. Although sarcoidosis has various clinical manifestations, its association with uveitis is more common in Japan than in other countries. The aim of this study was to investigate whether *TLR4* polymorphisms were associated with sarcoidosis-related uveitis in a Japanese population.

**Methods:**

Two hundred twenty-three patients with sarcoidosis and 206 healthy control subjects were recruited at seven sites in Japan. Eight single-nucleotide polymorphisms (SNPs) in *TLR4* were genotyped with a TaqMan assay, and allelic and phenotypic diversity were assessed in affected and control subjects.

**Results:**

We found no association with susceptibility to sarcoid-related uveitis for any of the SNPs analyzed. Strong linkage disequilibrium was observed among all the SNPs analyzed (D’≥0.78), which were located in one haplotype block.

**Conclusion:**

TLR4 polymorphisms do not play an important role in the development of uveitis in Japanese patients with sarcoidosis.

## Introduction

Sarcoidosis is a systemic inflammatory syndrome of unknown etiology characterized by the accumulation of immune effector cells in affected organs, such as lungs, lymph nodes, eyes, skin, and heart. The severity and mode of presentation are influenced by ethnicity. For instance, Löfgren’s syndrome, an acute form of sarcoidosis, is more common among Caucasians, and a more serious form of sarcoidosis has been observed in patients of African descent [1,2]. The incidence of ocular involvement is higher in Japan (50-93.5%) compared to that of other countries (25-60%) [2-8]. Clinically, ocular manifestations in sarcoidosis are considered bilateral, chronic, granulomatous uveitis [9]. The variety of clinical manifestations of sarcoidosis and the observed dependence on race and familial clustering suggest there may be a genetic predisposition for developing sarcoidosis [10-12]. Numerous studies have reported genetic associations between various major histocompatibility complex (MHC) genes and the susceptibility, severity, and presentation of sarcoidosis [13-17]. The first genome-wide search for genes that predispose for sarcoidosis in German families used microsatellite linkage analysis. These studies identified several chromosomal regions that contributed to the risk of sarcoidosis. The MHC genes displayed the most prominent signal in these regions. This supported the hypothesis that MHC genes were associated with the different manifestations of sarcoidosis [18]. Furthermore, another genome-wide association study that included >440,000 single-nucleotide polymorphisms (SNPs) in patients with sarcoidosis also reported a series of genetic associations [19].

It has been suggested that sarcoidosis results from exposure of genetically susceptible hosts to specific, but unidentified environmental agents [20]. The granuloma in sarcoidosis develops, in general, as a response to a persistent, weakly virulent antigen(s) that induces a local helper T cell type 1 (Th1) immune response [21].

Toll-like receptors (TLRs) are transmembrane proteins that function as pattern-recognition receptors (PRRs) in innate immunity. TLRs also provide a link to the activation of adaptive immunity by recognizing microbial components and inducing the production of cytokines, such as Interleukin 12 (IL12) and IL18 driving naïve T cells to differentiate into Th1 cells [22]. Among TLR family members, TLR4 is expressed in a wide variety of human cells, where it plays an important role in innate and adaptive immune responses to Gram-negative bacterial products, including lipopolysaccharides [22,23].

In recent studies, *TLR4* polymorphisms have been reported to be associated with various inflammatory diseases, including atherosclerosis, Crohn’s disease, ulcerative colitis, rheumatoid arthritis, prostate cancer, and Alzheimer’s disease [23-28]. These findings suggest that genetic variants of TLR4 that have abnormal ligand recognition properties may contribute to the development of various diseases, including sarcoidosis.

Due to its various clinical manifestations, sarcoidosis is often considered to be a family of disorders with clinically and genetically different phenotypes, rather than a single entity [29]. This study investigated whether *TLR4* polymorphisms were associated with susceptibility to sarcoidosis-related uveitis in Japanese patients.

## Methods

### Subjects

This study included 223 Japanese patients with sarcoidosis (52 men and 171 women) and 206 healthy Japanese controls (48 men and 158 women) from Yokohama City University, Hokkaido University, Fujita Health University, Tokyo University, Keio University, the Kumamoto City hospital, and the Yuasa Eye Clinic. All 223 patients had chronic sarcoidosis; 68 cases were clinically diagnosed and 155 cases were confirmed by biopsies. These 223 patients were divided into two groups: 196 (87.9%) with uveitis (the sarcoid-uveitis group) and 27 (12.1%) without uveitis (the sarcoid non-uveitis group). Clinical sarcoidosis was diagnosed according to the diagnostic criteria developed by the Japanese Society of Sarcoidosis and Other Granulomatous Disorders (JSSOG) in 1977 [9]. Uveitis with sarcoidosis was assessed based on *the Guidelines for Diagnosis of Ocular Lesions in Sarcoidosis* prepared by the JSSOG. The ocular features of sarcoidosis were defined as granulomatous uveitis plus two or more of the following: infiltration of the anterior chamber (mutton-fat keratic precipitates/iris nodules), trabecular meshwork nodules and/or tent-shaped peripheral anterior synechia, masses of vitreous opacities (snowball-like or string of pearls-like appearance), periphlebitis with perivascular nodules; multiple candle-wax type chorioretinal exudates and nodules, and/or laser photocoagulation spot-like chorioretinal atrophy [30].

All the control subjects were unrelated healthy volunteers that were ethnically similar to the patients. The study obtained the approval of the Ethics Committee of Yokohama City University School of Medicine, and was in compliance with the guidelines of the Declaration of Helsinki. Details of the study were explained to all patients and controls, and written informed consent was obtained for genetic screening.

### Analysis of *TLR4* polymorphisms

The QIAamp DNA Blood Maxi Kit (Qiagen, Tokyo, Japan) was used to extract genomic DNA from peripheral blood cells. Procedures were performed under standardized conditions to prevent variation in DNA quality.

The *TLR4* gene is located on chromosome 9q32-q33. We evaluated eight single-nucleotide polymorphisms (SNPs), including: rs10759930, rs1927914, rs1927911, rs12377632, rs2149356, rs11536889, rs7037117, and rs7045953 within *TLR4*. These SNPs were selected based on previous reports [31-33] and information from public sources, including the NCBI dbSNP, ABI, and HapMap databases. The selected SNPs had minor allele frequencies that were >5% (Table 1). Each SNP was approximately 1 to 5 kb long, and the set of SNPs spanned *TLR4*, from approximately 5 kb of the predicted 5’-untranslated region (UTR) to approximately 6 kb of the predicted 3’ UTR. Genotyping of the SNPs was performed with the TaqMan 5’ exonuclease assay using primers supplied by ABI (Foster City, CA). The probe emitted a fluorescent signal and was incorporated during the TaqMan Assay for Real-Time PCR (7500 Real Time PCR System; Applied Biosystems), following the manufacturer’s instructions.

**Table 1:** Allele frequencies of SNPs in the *TLR4* gene.

**dbSNP**	**Alleles**	**Position (bp)**	**Gene location**	**Minor allele frequency, n (%)**			**p (*SUvs.SN*)**	**p (*SUvs.C*)**	**p (*SNvs.C*)**
				**Sarcoid uveitis n=196**	**Sarcoid non-uveitis n=27**	**Controls n=206**			
rs10759930	T>C	119,501,442	5'-UTR	142 (36.2)	22 (40.7)	157 (38.1)	0.319	0.387	0.542
rs1927914	A>G	119,504,546	5'-UTR	141 (36.0)	22 (40.7)	158 (38.3)	0.295	0.274	0.577
rs1927911	G>A	119,509,875	Intron	137 (34.9)	21 (38.9)	159 (38.6)	0.398	0.097	0.945
rs12377632	C>T	119,512,551	Intron	139 (35.5)	21 (38.9)	157 (38.1)	0.458	0.228	0.857
rs2149356	G>T	119,514,020	Intron	137 (34.9)	21 (38.9)	158 (38.3)	0.398	0.123	0.901
rs11536889	G>C	119,517,952	3'-UTR	93 (23.7)	12 (22.2)	108 (26.2)	0.769	0.318	0.435
rs7037117	A>G	119,523,484	3'-UTR	87 (22.2)	15 (27.8)	89 (21.6)	0.275	0.811	0.225
rs7045953	A>G	119,525,616	3'-UTR	31 ( 7.9)	3 ( 5.6)	39 (9.5)	0.431*	0.410	0.430*

In the table, SNP represents single-nucleotide polymorphism, TLR4 represents toll-like receptor 4, and bp represents base pairs. Position is the distance from the short arm telomere. Gene location is the location of the SNP within the genomic *TLR4* sequence. P values were calculated with the χ^2^ test using a 2×2 contingency table. In the table, *SUvs.SN* represents sarcoid uveitis versus sarcoid non-uveitis, *SUvs.C* represents sarcoid uveitis versus control, and *SNvs.C* represents sarcoid non-uveitis versus control. The asterisk indicates calculation using Fisher's Exact Test.

### Statistical analysis

The Hardy-Weinberg equilibrium (HWE) was tested for each SNP in cases and controls. Differences in allele frequencies between cases and controls were assessed with the χ^2^ test or Fisher’s exact test. The Haploview 3.32 program was used to compute pairwise linkage disequilibrium (LD) statistics [34]. Standardized disequilibrium D’ and r^2^ were plotted. LD blocks were defined according to the criteria of Gabriel et al. [35]. Haplotype frequencies were estimated with an accelerated expectation-maximization algorithm, similar to the partition-ligation-expectation-maximization method described previously [36]. All p-values were derived from a 2-sided test, and p-values <0.05 were considered statistically significant.

## Results

**Figure 1:**
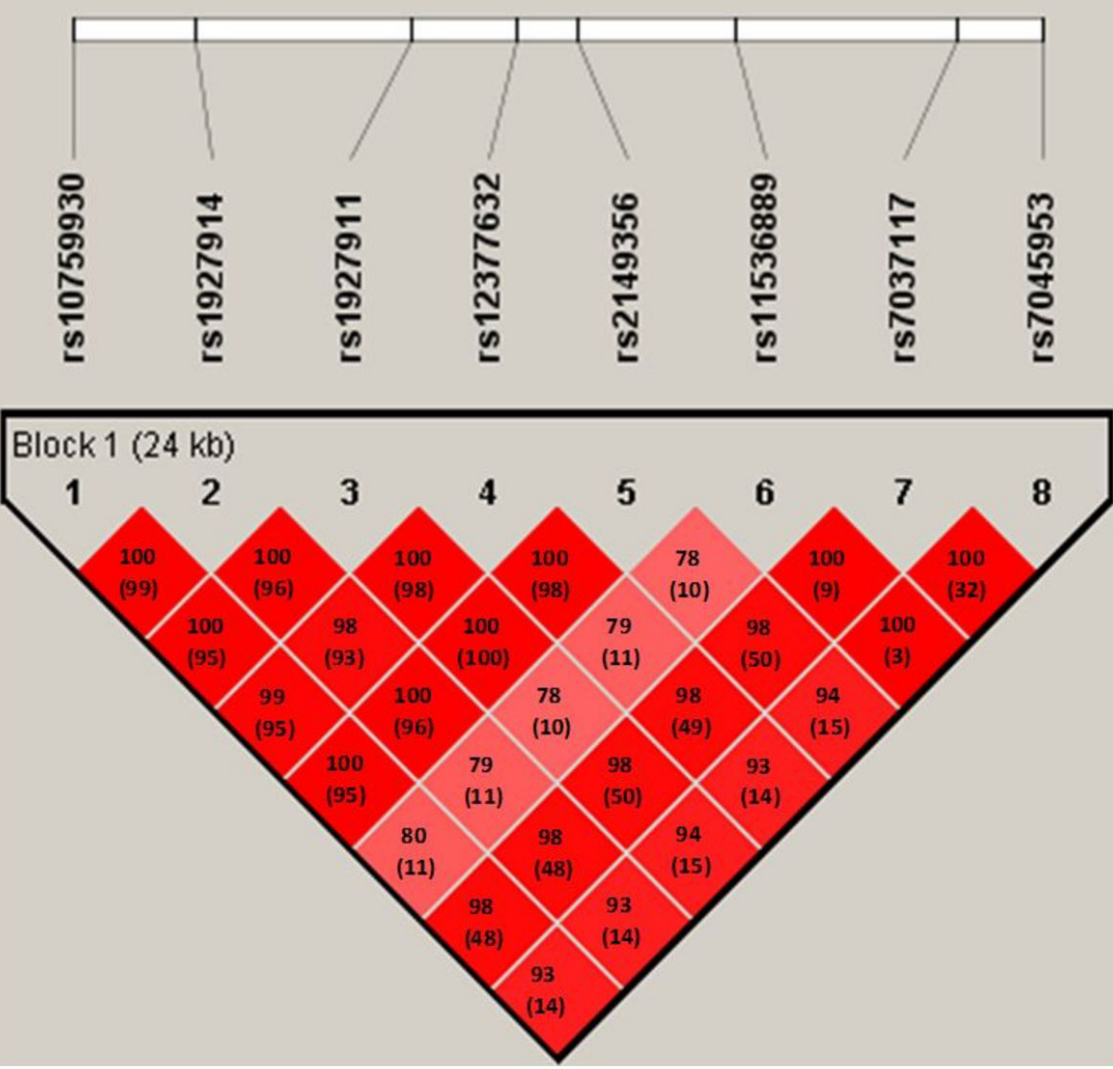
Structure of linkage disequilibrium (LD) plotted for eight SNPs in the *TLR4* gene. The D’ value and r^2^ value (in parentheses) that correspond to each SNP pair are expressed as a percentage and shown within the respective square. The gradient of D’ values (low to high values) is indicated by different shades of red (light to dark, respectively). The eight SNPs constitute a haplotype block that spans 24 kb of *TLR4*.

Eight SNPs in *TLR4* were genotyped. All SNPs were in HWE among both cases and controls (data not shown). All eight SNPs were located in one haplotype block, and the magnitude of LD between each SNP was extremely high, with pair-wise D’ values that were ≥0.78 (Figure 1).

Table 1 shows the details of the eight SNPs, including their genomic locations and minor allele frequencies in cases and controls. No statistically significant association was observed for any of the SNPs between the sarcoid uveitis group and the sarcoid non-uveitis group, between the sarcoid uveitis group and the control subjects, or between the sarcoid non-uveitis group and the control subjects (p≥0.05; Table 1).

The genotypic frequencies of the eight SNPs are shown in Table 2, Table 3, and Table 4. There were no differences in the minor allele frequency of each SNP between the sarcoid uveitis group and the sarcoid non-uveitis group, between the sarcoid uveitis group and the control subjects, or between the sarcoid non-uveitis group and the control subjects (p≥0.05; Table 2, Table 3, and Table 4).

**Table 2:** Genotype frequencies of eight SNPs in *TLR4*. Patients with sarcoid uveitis compared to those with sarcoid non-uveitis.

**dbSNP**	**Alleles (Maj/Min)**	**Subjects**	**Genotype frequency n (%)**				**Minor allele dominance (Maj/Min + Min/Min)**	
			**Maj/Maj**	**Maj/Min**	**Min/Min**	p (*SUvs.SN*)*	p (*SUvs.SN*)**	**OR (95%CI)**
rs10759930	T/C	SU	77 (39.3)	95 (48.5)	24 (12.2)	0.297	0.885	0.94 (0.41-2.14)
		SN	11 (40.7)	10 (37.0)	6 (22.2)			
rs1927914	A/G	SU	78 (39.8)	94 (48.0)	24 (12.2)	0.306	0.925	0.96 (0.42-2.18)
		SN	11 (40.7)	10 (37.0)	6 (22.2)			
rs1927911	G/A	SU	81 (41.3)	92 (46.9)	23 (11.7)	0.586	0.954	1.02 (0.45-2.32)
		SN	11 (40.7)	11 (40.7)	5 (18.5)			
rs12377632	C/T	SU	79 (40.3)	94 (48.0)	23 (11.7)	0.567	0.966	0.98 (0.43-2.23)
		SN	11 (40.7)	11 (40.7)	5 (18.5)			
rs2149356	G/T	SU	81 (41.3)	92 (46.9)	23 (11.7)	0.586	0.954	1.02 (0.45-2.32)
		SN	11 (40.7)	11 (40.7)	5 (18.5)			
rs11536889	G/C	SU	117 (59.7)	66 (33.7)	13 ( 6.6)	0.812	0.966	1.02 (0.45-2.31)
		SN	16 (59.3)	10 (37.0)	1 (3.7)			
rs7037117	A/G	SU	115 (58.7)	75 (38.3)	6 (3.1)	0.594	0.300	1.53 (0.68-3.43)
		SN	13 (48.1)	13 (48.1)	1 (3.7)			
rs7045953	A/G	SU	165 (84.2)	29 (14.8)	2 (1.0)	0.755	0.598	0.67 (0.19-2.35)
		SN	24 (88.9)	3 (11.1)	0 (0.0)			

In the table, SNP represents single-nucleotide polymorphism, TLR4 represents toll-like receptor 4, Maj represents major allele, Min represents minor allele, SU represents sarcoid uveitis, and SN represents sarcoid non-uveitis. The asterisk indicates the data was calculated with the χ^2^ test using a 3×2 contingency table. The double asterisk indicates the data was calculated with the χ^2^ test using a 2×2 contingency table.

**Table 3:** Genotype frequencies of eight SNPs in *TLR4*. Patients with sarcoid uveitis compared to control subjects.

**dbSNP**	**Alleles (Maj/Min)**	**Subjects**	**Genotype frequency n (%)**				**Minor allele dominance (Maj/Min + Min/Min)**	
			**Maj/Maj**	**Maj/Min**	**Min/Min**	p (*SUvs.C*)*	p (*SUvs.C*)**	**OR (95%CI)**
rs10759930	T/C	SU	77 (39.3)	95 (48.5)	24 (12.2)	0.493	0.915	0.98 (0.66-1.46)
		C	82 (39.8)	91 (44.2)	33 (16.0)			
rs1927914	A/G	SU	78 (39.8)	94 (48.0)	24 (12.2)	0.535	0.922	1.02 (0.72-2.44)
		C	81 (39.3)	92 (44.7)	33 (16.0)			
rs1927911	G/A	SU	81(41.3)	92 (46.9)	23 (11.7)	0.318	0.756	1.07 (0.72-1.59)
		C	82 (39.8)	89 (43.2)	35 (16.7)			
rs12377632	C/T	SU	79 (40.3)	94 (48.0)	23 (11.7)	0.440	0.919	1.02 (0.69-1.52)
		C	82 (39.8)	91 (44.2)	33 (16.0)			
rs2149356	G/T	SU	81 (41.3)	92 (46.9)	23 (11.7)	0.464	0.682	1.09 (0.78-2.65)
		C	81 (39.3)	92 (44.7)	33 (16.0)			
rs11536889	G/C	SU	117 (59.7)	66 (33.7)	13 ( 6.6)	0.617	0.327	1.22 (0.82-1.81)
		C	113 (54.9)	78 (37.9)	15 (7.3)			
rs7037117	A/G	SU	115 (58.7)	75 (38.3)	6 (3.1)	0.066	0.312	0.81 (0.54-1.21)
		C	13 (63.6)	61 (29.6)	14 (6.8)			
rs7045953	A/G	SU	165 (84.2)	29 (14.8)	2 (1.0)	0.582#	0.485	1.20 (0.72-2.03)
		C	168 (81.6)	37 (18.0)	1 (0.5)			

In the table, SNP represents single-nucleotide polymorphism, TLR4 represents toll-like receptor 4, Maj represents major allele, Min represents minor allele, SU represents sarcoid uveitis, and C represents control subjects. The asterisk indicates the data was calculated with the χ^2^ test using a 3×2 contingency table. The double asterisk indicates the data was calculated with the χ^2^ test using a 2×2 contingency table. The sharp (hash mark) indicates the data was calculated with Fisher's Exact Test.

**Table 4:** Genotype frequencies of eight SNPs in *TLR4*. Patients with Sarcoid non-uveitis compared to control subjects.

**dbSNP**	**Alleles (Maj/Min)**	**Subjects**	**Genotype frequency n (%)**				**Minor allele dominance (Maj/Min + Min/Min)**	
			**Maj/Maj**	**Maj/Min**	**Min/Min**	p (*SNvs.C*)*	p (*SNvs.C*)**	**OR (95%CI)**
rs10759930	T/C	SN	11 (40.7)	10 (37.0)	6 (22.2)	0.659	0.926	1.04 (0.46-2.35)
		C	82 (39.8)	91 (44.2)	33 (16.0)			
rs1927914	A/G	SN	11 (40.7)	10 (37.0)	6 (22.2)	0.645	0.887	1.06 (0.47-2.40)
		C	81 (39.3)	92 (44.7)	33 (16.0)			
rs1927911	G/A	SN	11 (40.7)	11 (40.7)	5 (18.5)	0.965	0.926	1.04 (0.46-2.35)
		C	82 (39.8)	89 (43.2)	35 (16.7)			
rs12377632	C/T	SN	11 (40.7)	11 (40.7)	5 (18.5)	0.923	0.926	1.04 (0.46-2.35)
		C	82 (39.8)	91 (44.2)	33 (16.0)			
rs2149356	G/T	SN	11 (40.7)	11 (40.7)	5 (18.5)	0.911	0.887	1.06 (0.47-2.40)
		C	81 (39.3)	92 (44.7)	33 (16.0)			
rs11536889	G/C	SN	16 (59.3)	10 (37.0)	1 (3.7)	0.766	0.665	1.20 (0.53-2.71)
		C	113 (54.9)	78 (37.9)	15 (7.3)			
rs7037117	A/G	SN	13 (48.1)	13 (48.1)	1 (3.7)	0.145	0.120	0.53 (0.24-1.19)
		C	13 (63.6)	61 (29.6)	14 (6.8)			
rs7045953	A/G	SN	24 (88.9)	3 (11.1)	0 (0.0)	0.625	0.347	1.81 (0.52-6.32)
		C	168 (81.6)	37 (18.0)	1 (0.5)			

In the table, SNP represents single-nucleotide polymorphism, TLR4 represents toll-like receptor 4, Maj represents major allele, Min represents minor allele, SN represents sarcoid non-uveitis, and C represents control subjects. The asterisk indicates the data was calculated with the χ^2^ test using a 3×2 contingency table. The double asterisk indicates the data was calculated with the χ^2^ test using a 2×2 contingency table.

Furthermore, there were no significant differences in the haplotype frequencies of the eight SNPs between the sarcoid uveitis group and the sarcoid non-uveitis group, between the sarcoid uveitis group and the control subjects, or between the sarcoid non-uveitis group and the control subjects (p≥0.05; Table 5)

**Table 5:** Haplotype frequencies of SNPs in *TLR4*.

**Haplotype**	**Frequency, n (%)**			**p (*SUvs.SN*)**	**p (*SUvs.C*)**	**p (*SNvs.C*)**
	**Sarcoid uveitis n=196**	**Sarcoid non-uveitis n=27**	**Controls n=206**			
TAGCGGAA	80 (40.8)	10 (37.0)	75 (36.4)	0.707	0.364	0.949
TAGCGCAA	43 (21.9)	6 (22.2)	50 (24.3)	0.973	0.579	0.815
CGATTGAA	23 (11.7)	3 (11.1)	32 (15.5)	1.000*	0.268	0.593*
CGATTGGA	27 (13.8)	6 (22.2)	25 (12.1)	0.247	0.624	0.147
CGATTGGG	16 (8.2)	2 (7.4)	20 (9.7)	1.000*	0.588	0.757*
CGATTCAA	3 (1.5)	0 (0.0)	2 (1.0)	1.000*	0.678*	1.000*

In the table, SNP represents single-nucleotide polymorphism and TLR4 represents toll-like receptor 4. Position is the distance from the short arm telomere. Haplotypes with frequency less than 1% are not listed. P values were calculated with the χ^2^ test using a 2×2 contingency table. In the table, *SUvs.SN* represents sarcoid uveitis versus sarcoid non-uveitis, *SUvs.C* represents sarcoid uveitis versus control, and *SNvs.C* represents sarcoid non-uveitis versus control. The asterisk indicates calculation using Fisher's Exact Test.

## Discussion

In this study, we investigated whether there were any associations between *TLR4* polymorphisms and uveitis with sarcoidosis. We found no associations for any of the SNPs analyzed. Several infectious organisms, including mycobacteria, mycoplasma, propionibacteria, and cytomegalovirus, have been proposed as potential causes of sarcoidosis [20,37,38]. The mycobacterium tuberculosis heat shock proteins (Mtb-HSPs) have also been suggested as causative agents of sarcoidosis based on the cross-reactivity observed between mycobacterial and human HSPs [39,40]. A SNP within the *HSP-70/Hom* gene was identified as a factor associated with susceptibility to sarcoidosis [41]. Others have also reported that SNPs in *HSP-70/1* and *HSP-70/Hom* were associated with sarcoid-related uveitis [29]. TLR4 recognizes endogenous ligands, including heat shock proteins (HSP60, HSP70) and exogenous ligands; upon binding these ligands, TLR4 induces the production of proinflammatory cytokines [22,42,43]. Therefore, it was hypothesized that TLR4 and its ligands would be implicated in the development of sarcoidosis. The results of this study do not support that hypothesis.

The long arm of chromosome 9, which harbors *TLR4*, was identified as a region related to the susceptibility to sarcoidosis by genome-wide microsatellite linkage analysis in a German population [18]. This suggested that *TLR4* polymorphisms were associated with sarcoidosis. Two different SNPs in *TLR4*, rs4986790 (Asp299Gly) and rs4986791 (Thr399Ile), affected the extracellular domain of the TLR4 receptor. These were reported to be associated with the development of chronic sarcoidosis in a German case control study [44]. However, the results could not be replicated in Dutch and Greek populations [45,46]. Previously, we analyzed the same SNPs in *TLR4* that were examined in this study to determine whether they were associated with susceptibility to Behçet's disease (BD) and normal tension glaucoma [31,32]. In that study, we found that the SNP, rs7037117, located in the 3’-untranslated region, was significantly associated with BD and normal tension glaucoma. We also explored the same SNPs to determine associations to BD in a Korean population. We found the most frequent occurring haplotype was significantly increased in patients with BD that were positive for HLA-B*51 and in patients with BD and arthritis [33]. It was also reported that two non-synonymous *TLR4* SNP sites, Asp299Gly (rs4986790) and Thr399Ile (rs4986791), were associated with elevated serum cytokines in a Caucasian population [26]. However, no studies have detected these non-synonymous mutations in Asian populations, including Koreans [47]. Thus, we did not examine those two non-synonymous SNPs because they were monomorphic in a Japanese population. Our results showed no differences in allelic diversity among patients that had sarcoidosis with or without uveitis and healthy control subjects. However, our results could not completely rule out the possibility that the TLR4 signaling pathway plays a role in the development of uveitis in patients with sarcoidosis. Although the discrepancy between our results and those of the German study on chronic sarcoidosis might be explained by racial differences, it will be necessary to confirm this negative association in future studies with larger cohorts.

In conclusion, the present study showed that *TLR4* polymorphisms were not significantly associated to the development of uveitis with sarcoidosis in the Japanese population.
